# Beyond PTSD and Fear-Based Conditioning: Anger-Related Responses Following Experiences of Forced Migration—A Systematic Review

**DOI:** 10.3389/fpsyg.2018.02592

**Published:** 2018-12-19

**Authors:** Martti T. Tuomisto, Jane E. Roche

**Affiliations:** ^1^Faculty of Social Sciences (Psychology), University of Tampere, Tampere, Finland; ^2^Faculty of Social Sciences, University of Tampere, Tampere, Finland

**Keywords:** anger, grief, interpersonal relations, refugees, stress disorders, post-traumatic

## Abstract

**Introduction:** Experiences of forced migration include traumas that are interpersonal in nature, as well as ongoing emotional responses, stress, and frustration in post-migration setti ngs. Open questions exist, regarding anger/anger-like responses following experiences of persecution and ongoing stress. The aim of this study was to explore the adaptive and maladaptive underlying mechanisms of anger/anger-like responses, cultural, linguistic, and social contingencies, and possible interventions for problematic anger behavior.

**Method:** We searched two databases (PsycINFO and PILOTS) with the following search terms: (refugee OR “asylum seek^*^” OR IDP OR “internal^*^ displac^*^” OR “forced migra^*^” OR “involuntary migra^*^”) AND anger.

**Findings:** This search yielded 34 studies that were included in the final review. Although, anger is a moral, adaptive, and prosocial response, dysfunctional anger/anger-like responses arise from PTSD, “moral injury,” complicated grief, and independent forms of anger behavior. Cultural, linguistic, and social issues also emerged from the search. Finally, considerations for treatment and intervention are discussed.

**Discussion:** Anger responses following experiences of forced migration may require assessment beyond PTSD models currently framed by DSM and ICD. A very promising framework is the Adaptation and Development after Persecution and Trauma (ADAPT) model.

**Implications:** Further longitudinal and epidemiological research will be necessary to continue testing the ADAPT model and to begin the process of assessing its cross-cultural coherence in other refugee populations (e.g., see Hinton et al., 2003). As anger behavior is also a societal issue, avenues for reconciliation, expression of grievances, employment, civic participation, and integration are needed.

## Beyond PTSD and Fear-Based Conditioning: Anger-Related Responses Following Experiences of Forced Migration – A Systematic Review

Approximately 68.8 million people have been forced to escape their homes due to recent conflicts around the world (UNHCR, 2015). Exposed to life threatening situations, multiple losses (Silove, 1999; Rees and Silove, 2011), and persecution (UN General Assembly, 1951), the quality of life and mental health of refugees^1^ may be compromised (Silove, 1999; Silove et al., 2000; Cohen, 2008; Meyer, 2013) and further compounded by post-migratory stressors, including marginalization, acculturation problems, poverty, or stressful asylum procedures (cf. Silove, 1999; Meffert et al., 2010; Rousseau and Foxen, 2010).

A prevalent area of study for refugees is post-traumatic stress disorder (PTSD), the framework of which, in recent years, has gone through a number of changes in both the Diagnostic Statistical Manual (American Psychiatric Association, 2013) and the International Classification of Diseases (ICD; Friedman, 2013, 2014). Under the traditional fear-based trauma model, adaptive anger responses (e.g., irritability, aggressive behavior) indicate physiological reactions to aversive stimuli (i.e., arousal; Chemtob et al., 1997), but become conditioned anxiety responses to reminders of the traumatic event(s). No longer designated as an anxiety disorder, however, the DSM-5 PTSD model now accounts for higher-order verbal processes mediated by complex social behavior and contexts, in accordance with modern learning theory (Zoellner et al., 2011; Salcioglu and Başoglu, 2013; Friedman, 2014).

The ICD-11 will also identify these changes in a new category called “Complex PTSD” (Cloitre et al., 2013; Friedman, 2014). Accordingly, human-instigated trauma potentially alters the survivor's long-term social behavior and fundamental thoughts about the world and oneself (Doerr-Zegers et al., 1992). Persistent, complex anger behavior often elicits such alterations (Bryant and Nickerson, 2013; cf. Silove, 1996) and problems with managing emotional responses (Nickerson et al., 2015a).

Despite controversy and arguments that PTSD should remain an anxiety disorder (Zoellner et al., 2011), these shifts in the field represent well-intentioned efforts to adapt to transnational, albeit morally and politically charged issues, reflecting Başoglu's (2001) call for more research on the changes in emotions and thoughts that follow experiences of mass injustice. However, (Bensimon et al., 2013) argue that the new models fail to reflect the complex, inhumane, and national character of forced migration-related traumas; distinct, not only in their threat to survival, but also in their other forms of uncontrollability (cf. Seligman, 1975; Silove, 1996; Başoglu, 2001; Bryant and Nickerson, 2013). Studies on Holocaust survivors (Danieli, 1998) and American war veterans (Koffel et al., 2012) indicate anger responses may be more fundamental to such experiences. Post-migration contexts may also worsen (Brooks et al., 2011; Rees et al., 2013) or lead to the development of anger responses independent from anxious and depressive behavior (Zarowsky, 2004; Silove, 2007; Nickerson et al., 2015a).

The overarching themes emerging from our search dealt with experiences of injustice, repeated, human-instigated incidents of trauma, and subsequent anger/anger-related responses. Moral issues (Summerfield, 2003; Zarowsky, 2004), as well as protective and survival functions of anger behavior, are regarded as essential (Kanninen et al., 2003; Kim et al., 2013). Most often, research highlights anger/anger-related responses in relationship to PTSD (e.g., see Abe et al., 1994; Hinton et al., 2009; Bryant and Nickerson, 2013), often exposing shortcomings in the conventional trauma model (e.g., see Bryant and Nickerson, 2013; Nickerson et al., 2015b). Anger responses also fall under other frameworks including “moral injury” (i.e., the lifelong effects of bearing witness to acts that compromise moral principles and expectations; Litz et al., 2009) and complicated grief (Tay et al., 2015a). Anger responses independent from anxiety, moral injury, and grief are also explored (Silove et al., 2009; Brooks et al., 2011; Tay et al., 2015a). Additionally, several studies highlight the importance of validating and properly translating measurements when studying anger/anger-related responses across refugee populations (e.g., see Ekblad et al., 2002; Summerfield, 2003; Silove et al., 2009; Meffert et al., 2010; Brooks et al., 2011; Rees and Silove, 2011; Liddell et al., 2013; Tay et al., 2015b). Notwithstanding adverse emotional and social behavior (e.g., see Mandic and Mihaljevic, 1993; Başoglu et al., 2005; Hinton et al., 2009; Meffert et al., 2014), anger/anger-related responses can be prosocial when individuals are connected to their social causes (e.g., campaigning for restitution or rights; Zarowsky, 2004; Kira et al., 2009; Rees and Silove, 2011).

Our purpose was to conduct a literature review and focus specifically on anger/anger-like responses that follow experiences of forced migration. To our knowledge, this is the first review on this particular topic. The aim of this study was to explore the complexity of responses to traumatic situations in the context of forced migration, particularly, the relationship between PTSD and anger responses. A central question for this study was this: are anger responses symptoms of PTSD or are they independent? Another important question is the following: in what cases is anger independent from PTSD and why? Exploring these questions has implications for diagnosis and treatment. Deepening scientific understanding on this front will broaden and enrich responses and treatment options for the increasingly large flows of people forced to flee situations characterized by injustice.

## Methods

### Inclusion Criteria

We conducted a systematic review and studied samples of people forced to migrate within or outside the borders of their home countries due to war, conflict, persecution, marginalization, poverty, or other humanitarian disaster. These samples originate from a wide range of involuntary migrant populations, including refugees, asylum seekers,^2^ failed asylum seekers, IDPs,^3^ or those with other forms of humanitarian protection (e.g., Subsidiary Protection; Temporary Humanitarian Protection).

### Information Sources

We used two databases (PsycINFO and PILOTS) between 1979 and 2016 with the following search terms: (refugee OR “asylum seek^*^” OR IDP OR “internal^*^ displac^*^” OR “forced migra^*^” OR “involuntary migra^*^”) AND anger.

### Selection Process

**Figure 1:**
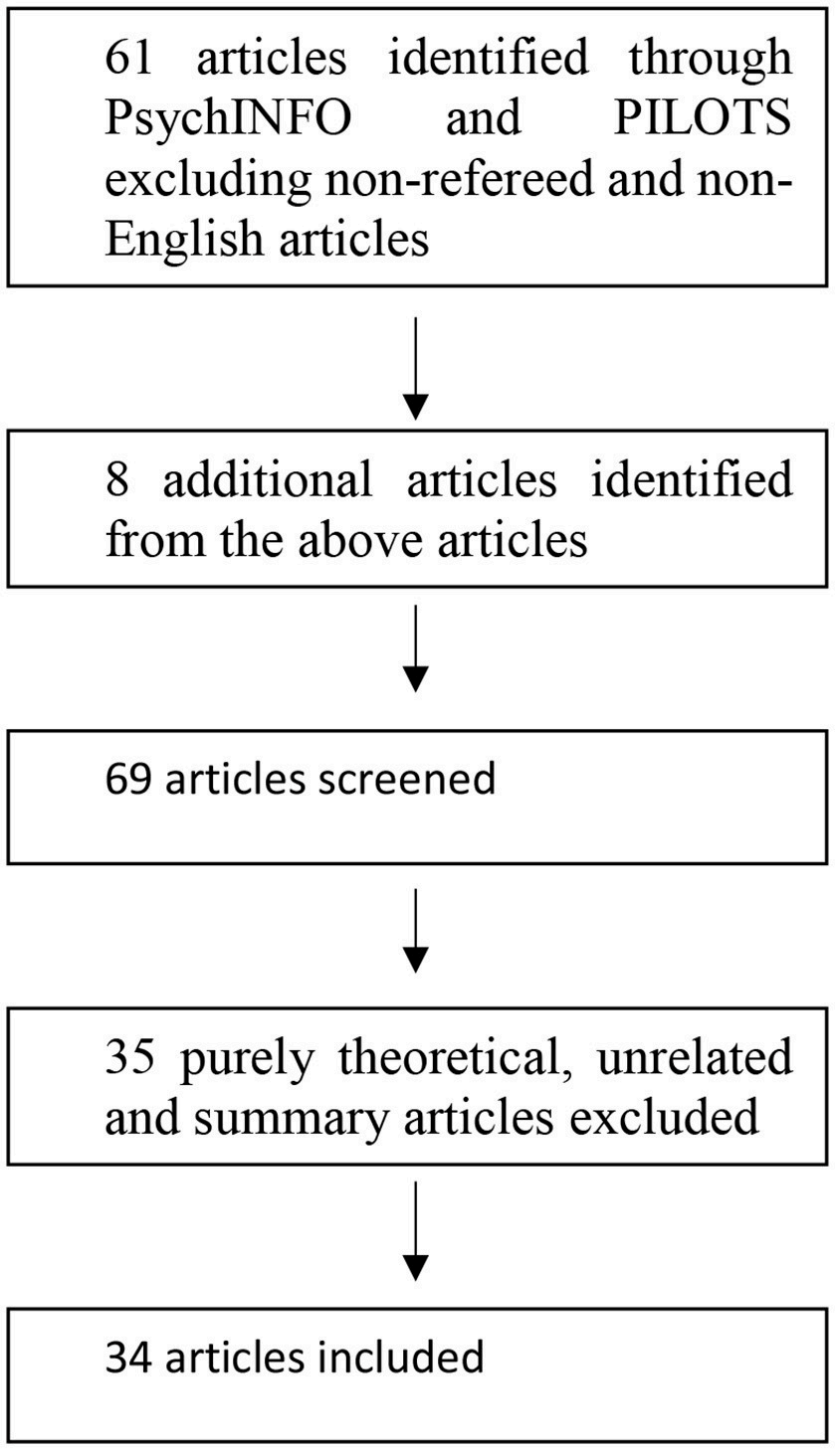
Flow chart of the selection of studies.

This search yielded 61 different articles (see Figure 1). Between September 2015 and May 2016, two independent readers facilitated the selection of papers that were in English. Papers were eligible if the study's aims included assessing anger-related phenomena or had findings or implications about anger-related phenomena. In addition, papers were eligible if participants fit the above definition(s) of forced migrants. To screen them for scientific rigor, included articles had to have an empirical basis (i.e., quantitative, qualitative, or mixed methods). However, we also included some clinical case studies to explore treatment recommendations. To screen for quality, we included only peer-reviewed articles. In the final review, 34 studies were included. Omitted studies were purely theoretical in nature, unrelated to the topic, were not peer-reviewed, or were only summaries of empirical issues.

### Data Extraction Process

To facilitate the data extraction process, two independent researchers divided included papers into qualitative, quantitative, mixed methods, and clinical case studies. A template was used to record aims, methods, findings, and implications of each study. We compared and contrasted studies to one another and to theoretical and treatment literature. Our aim was to reduce the risk of bias of the conclusions in this way. To create a synthesis, we extracted common empirical themes as indicated in the titles of the article and questions that arose from the process. The topics covered in the review are presented in the Table 1.

**Table 1:** Information covered in the systematic review: main topics and subtopics.

Definitions of anger/anger-related behavior
Moral anger
Adaptive and protective functions of anger behavior
Post-traumatic anger responses Conceptual issues in the DSM and ICD
Relationship between PTSD and anger behavior
Normative and complicated bereavement
Post-migration stressors
The Adaptation and Development after Persecution and Trauma model
An independent framework for anger responses
Cultural and conceptual issues Tools for cross-cultural coherence
Ethno-behavior science
Ethno-physiology
Social consequences
Health factors
Current treatments and interventions Behavioral interventions
Complicated grief
Cultural sensitivity
Systems-based and multidisciplinary approaches

## Results

### Definitions of Anger/Anger-Related Behavior

Although universally experienced, anger is the most contextually variable emotion, which makes it difficult to define and diagnose (Eckhardt and Deffenbacher, 1995; DiGiuseppe and Tafrate, 2007). In fact, only one anger diagnosis exists, called intermittent explosive disorder (IED; see later), and even it may not be regarded as pure anger diagnosis. Lang (1968) offers a model of three response systems (i.e., verbal, overt-motor, and physiological responses) that could be useful in understanding anger behavior. Other experts define anger as a primarily affective, but also cognitive (i.e., relating to thoughts or ideas about one's internal experience and external environment), biobehavioral, and sensory-motor (Novaco, 1986; Spielberger, 1999; DiGiuseppe and Tafrate, 2007) response to a self-reported injustice (Weiss et al., 2003), “frustration” (Berkowitz and Harmon-Jones, 2004), or threat (DiGiuseppe and Tafrate, 2007). Like other emotions, anger behavior may involve unlearned biobehavioral responses, respondent responses, or verbally mediated responses to other feelings, actions, social behavior, rules, and complex environmental factors (DiGiuseppe and Tafrate, 2007; see also Schacter and Singer, 1963; Tavris, 1989).

One recurring challenge in emotion theory arises in how to locate “anger-like” responses (e.g., hostile aggression, irritability, or paranoid behavior) along the wider spectrum of anger behavior (Berkowitz and Harmon-Jones, 2004). For example, irritability is defined as a primarily biobehavioral response that undermines an individual's ability to tolerate traumatic events and conditions threatening social networks and values (DiGiuseppe and Tafrate, 2007). Hostile behavior, which shows conceptual overlap with “trait” anger (Spielberger, 1999; Caroll, 2013), is defined as a learned, persistent tendency to respond with angry feelings, thoughts, or actions toward particular people, things, or ideas (DiGiuseppe and Tafrate, 2007; Smith et al., 2013). In addition, paranoid behavior may also lead to or include anger responses involving chronic thoughts and feelings that others are hostile to oneself (Tarrier and Taylor, 2014). However, some emotion theorists (e.g., Clore et al., 1993) contend that these anger-related behaviors do not constitute pure emotionality (i.e., unlearned or respondent behavior) because they involve operant responses. Aggressive behavior, also conceptually problematic, involves a wide range of both prosocial and antagonistic actions, from constructive verbal reproach to direct violence (Wolman, 1989). However, DiGiuseppe and Tafrate (2007) note that violence, mediated by a number of contingencies, does not necessarily result from an anger response [e.g., see “instrumental aggression” (Buss, 1971)]. A useful perspective that acknowledges the range and subtleties of adaptive and problematic anger-like behavior, but also discerns from unrelated responses (e.g., instrumental aggression), is that of social contingencies strengthening or weakening the behavior (Skinner, 1971), and the concurrent emotional or sensory responses seen as qualities of the behavior contingency. This view may also help us to address Euro-American biases (Summerfield, 2003; Zarowsky, 2004), especially amid complex, transnational contexts involving mass human rights violations (Silove, 2013).

### Moral Anger

Anger behavior has a social function (Tavris, 1989; DiGiuseppe and Tafrate, 2007); therefore, it is best explored among refugees from both psychiatric and value-based perspectives (e.g., see Summerfield, 2003; Zarowsky, 2004; Meffert et al., 2010; Rousseau and Foxen, 2010). For instance, studies highlight a justifiable tendency among participants to fixate on perpetrators of persecutory experiences that occurred before flight (for example, see Abe et al., 1994; Rees and Silove, 2011; Bryant and Nickerson, 2013). In the post-migration context, anger responses (i.e., high “trait anger”) among Sudanese refugees with PTSD (*n* = 22) were significantly correlated with reasonable and understandable feelings of injustice and betrayal due to illegal and abusive treatment by an institution in Egypt that is intended to help refugees (Meffert et al., 2010).

### Adaptive and Protective Functions of Anger Behavior

Ideally, anger responses also help individuals survive danger and cope with adverse conditions (Tavris, 1989; DiGiuseppe and Tafrate, 2007). For instance, Kim et al. (2013) determined in a sample of North Korean refugee women (*n* = 2,163) that those exhibiting paranoid and hostile behavior had been exposed to the severest and highest number of forced migration-related traumatic events. As causal inferences cannot be inferred from the study, it is unclear if these social behaviors were acquired as a result of these events or if they had been formed during their earlier life, possibly preparing the women for later incidents of trauma-related problems. Other evidence indicates that social anger behavior may function as avoidance behavior to protect against the development of PTSD-type responses (cf. Kanninen et al., 2003). For example, participants in a sample of Palestinian torture survivors and refugees (*n* = 176), who exhibited anger-related “character traits” acquired during abusive childhoods, were least likely to develop certain post-traumatic stress responses (Kanninen et al., 2003). This supports the view that anger-related contingencies are biologically more important than anxiety related contingencies in general.

### Post-traumatic Anger Responses

#### Conceptual Issues in the DSM and ICD

Başoglu (2001) theorizes that the “sense of injustice” that develops because of war- and conflict-related experiences potentially leads to adverse, long-term changes in thoughts and emotions about the world. The need to index this “sense of injustice” first became clear in a study on Southeast Asian refugees (*n* = 308) by Abe et al. (1994), who found that anger responses (i.e., anger toward the perpetrator) were what most significantly distinguished participants without PTSD from participants with it. It was deemed necessary to add an Anger Reaction Index to the research design of the study because the DSM-IV model of PTSD failed to account for appropriate anger-associated criteria, including those with a clear verbal basis. This shortcoming may have reflected conceptual bias in the earlier fear-based PTSD model (cf. Van de Vijver and Tanzer, 2004).

Other studies also reveal conceptual problems in DSM-IV (e.g., see Momartin et al., 2002; Hinton et al., 2003; Momartin and Coello, 2006; Charney and Keane, 2007; Bryant and Nickerson, 2013; Draijer and Van Zon, 2013). For instance, Momartin et al. (2002) explored trauma experiences among 126 Bosnian refugees resettled in Australia and discovered that anger responses (i.e., feelings of betrayal, distrust, and frustration) emerged from autobiographical accounts. They claimed that such conceptual issues are too abstract to be identified by DSM-IV criteria.

Shortcomings have also been observed with regard to the ICD-10 (Momartin and Coello, 2006; Bryant and Nickerson, 2013). For instance, a clinical case study of an Iraqi torture survivor revealed that the fear-based PTSD model was able to account for the client's anger-related arousal responses, but failed to encompass his persistent, negative thoughts and feelings and his tendency to fixate on past injustices (Bryant and Nickerson, 2013). Bryant and Nickerson (2013) determined that these thoughts and tendencies were not anxiety responses, but could be understood as anger-related changes in the client's long-term social behavior following ongoing exposure to mistreatment and betrayal in Iraq. Correspondingly, Momartin and Coello (2006) proposed in their clinical case study that their client's anger responses were not only dysfunctional, biobehavioral reactions. Rather, chronic anger behavior resulted from long-lasting changes in social behavior following experiences of torture. Accordingly, clients in both of these case studies were diagnosed with “Complex PTSD” (Momartin and Coello, 2006; Bryant and Nickerson, 2013).

The DSM 5 has also made changes that seemingly encompass trauma to a more complete extent (American Psychiatric Association, 2013; cf. Friedman, 2013; cf. 2014), adapting Criterion D to account for changes in social behavior and feelings and thoughts about the world (Friedman et al., 2011; Friedman, 2013, 2014; Levin et al., 2014). However, Nickerson et al. (2015b) suggest that these changes result from moral injury, a concept that is more extensive than what the DSM 5 model of PTSD offers. They found that it explained 10% of the variance in anger behavior in a clinical sample (*n* = 134) of resettled refugees and asylum seekers in Switzerland, thereby suggesting that it partially accounts for the presence of anger responses.

#### Relationship Between PTSD and Anger Behavior

Studies also diverge with regard to the precise relationship between PTSD and anger behavior. For instance, Hinton's (2003) research on Cambodian refugees exhibited that anger responses acted as antecedents for triggering trauma memories among participants (in this case, slavery during the Khmer Rouge Regime). They hypothesized that participants remembered this particular trauma because of socially mediated, classically (respondently) conditioned, and biobehavioral anger behavior.

Other researchers posit that anger responses are avoidance behaviors (Charney and Keane, 2007; Draijer and Van Zon, 2013). For instance, Draijer and Van Zon (2013) proposed in their clinical case study that anger responses are forms of escape behavior that may lead to dissociation, when their client, a former Sierra Leonean child soldier resettled in the Netherlands, exhibited aggressive behavior as a means to avoid stimuli eliciting feelings of shame. Similarly, Charney and Keane's (2007) study revealed that anger responses among a sample of Bosnian refugees (*n* = 115) were associated with both arousal and avoidance behavior.

### Normative or Complicated Bereavement?

The process of bereavement commonly involves anger behavior (Kübler-Ross, 1969). However, prolonged grief may involve ruminative behavior (Morina, 2011). Some researchers suggest that the inhumane nature of forced migration-related losses disrupts the grieving process (Tay et al., 2015a). For instance, in evaluating the cross-cultural adequacy of DSM 5 and forthcoming ICD complicated grief models among West Papuan refugees (*n* = 230) in Papua New Guinea, a survey-based study revealed that complicated grief diverged from western models, being predicted by anger behavior arising from a chronic “sense of injustice” (Tay et al., 2015a). Similar findings emerged in Pirta's (2014) study on IDPs of Bhakra Dam in India, who were still grieving the loss of their homes from 50 years earlier. The authors proposed that anger responses elicited memories of home and prolonged bereavement.

### Post-migration Stressors

In addition to experiences of persecution, refugees are frequently exposed to disempowering, unjust post-migration settings (Westermeyer and Uecker, 1997; Sedighdeilami, 2004; Silove et al., 2009; Meffert et al., 2010; Brooks et al., 2011). Accordingly, the relationship between post-migration conditions and maladaptive anger/anger-related responses has been explored (Lin et al., 1979; Silove, 1996; Westermeyer and Uecker, 1997; Ekblad et al., 2002; Sedighdeilami, 2004; Silove et al., 2009; Meffert et al., 2010; Brooks et al., 2011; Bryant and Nickerson, 2013). An earlier study on Vietnamese refugees (*n* = 293) in the USA, for example, signified that post-migration problems were associated with an increase in anger responses among participants (Lin et al., 1979). Additionally, the above investigation on Sudanese refugees revealed a significant correlation between high “trait anger” and experiences of institutional discrimination in the Egyptian asylum system (Meffert et al., 2010). Similarly, the above case study on the Iraqi torture survivor revealed that his problematic anger responses derived from his fixation on ongoing stressors, in addition to past trauma (Bryant and Nickerson, 2013).

Other studies implicate societal ostracism as a potential source of problematic anger responses (Ekblad et al., 2002; Sedighdeilami, 2004). For example, a 10 years longitudinal study on the psychological impact of pre- and post-migration variables on Hmong refugees (*n* = 102) in the USA revealed that hostile responses were predicted by stimuli eliciting feelings of alienation as a motivational variable, especially during the 1st year of resettlement (Westermeyer and Uecker, 1997). Likewise, Ekblad et al. (2002) proposed that hostile behavior in the above Kosovan sample (*n* = 131) arose not only from experiences of torture, but also from the decade of marginalization the community had endured after flight. Similarly, self-reported discrimination was associated with anger behavior among 205 Iranian refugees resettled in Canada (Sedighdeilami, 2004).

### The Adaptation and Development After Persecution and Trauma (ADAPT) Model

Several authors hypothesize that the combined effect of pre-migration trauma and post-migration socio-economic variables mediates the behavioral pathway to anger behavior among forced migrants (Silove et al., 2009; Brooks et al., 2011; Nickerson et al., 2015a). In exploring this particular relationship, Silove et al. (2009) tested the Adaptation and Development after Persecution and Trauma (ADAPT) model on an epidemiological sample of Timorese participants (*n* = 1,247), a community affected by mass displacement due to past Indonesian occupation, determining that over one quarter of those exhibiting problematic anger responses was a group of men unable to find employment. They later established that the relationship between human-instigated trauma and anger responses was mediated by ongoing hardship (e.g., unemployment, poor education, discrimination) in the post-conflict context (Brooks et al., 2011). The ADAPT model has also been refined by recent cross-sectional research demonstrating that problems with managing emotional behavior accounts for these composite effects (Nickerson et al., 2015a).

### An Independent Framework for Anger Responses?

Both concurrent and independent anger responses have been explored (e.g., see Westermeyer and Uecker, 1997; Silove et al., 2009; Meffert et al., 2010; Brooks et al., 2011). For example, the above study on Hmong refugees revealed that chronic hostile behavior was significantly correlated with depression symptoms that developed during early stages of resettlement (Westermeyer and Uecker, 1997). However, according to other evidence, anger behaviors are independent responses (Silove et al., 2009; Brooks et al., 2011). For instance, the above studies on Timorese IDPs found that approximately 40% of the community met criteria for problematic anger behavior (Silove et al., 2009; Brooks et al., 2011). However, <10% of those exhibiting problematic anger behaviors had PTSD, while ~5% showed symptoms for anxiety and depression, thereby reinforcing the hypothesis that anger responses require independent frameworks for investigation.

Although Silove et al. (2009) hypothesized that anger behavior in the Timorese community persisted after other post-traumatic stress responses had subsided; they determined that the combined effect of injustice and later socioeconomic stressors caused anger behavior to develop independently. A similar issue arose in the above study on Sudanese refugees (Meffert et al., 2010). Although Meffert et al. theorized that anger was a response to experiences of pre-flight trauma they inferred an insignificant correlation. Instead, they concluded that the unjust actions of the UNHCR were more strongly associated with high “trait” anger behavior. Momartin and Coello (2006) also proposed in the above case study that, because the client had suppressed anger responses for fear of punishment during imprisonment, covert anger behavior developed independently.

### Cultural and Conceptual Issues

#### Tools for Cross-Cultural Coherence

Research designs inappropriate for refugee populations may produce bias (e.g., response, item, and conceptual bias; cf. Van de Vijver and Tanzer, 2004). Assessments such as the State-Trait Anger Expression Inventory (STAXI) have been validated in few refugee populations. To develop appropriate and precise psychometric measures and to increase cultural competency and awareness of social variables that may influence how respondents evaluate others' actions and the world around them (cf. Betancourt et al., 2003, and Hinton et al., 2012), various studies have utilized “bottom up” methods such as community consultation (e.g., see Westermeyer and Uecker, 1997; Silove et al., 2009; Meffert et al., 2010; Brooks et al., 2011; Rees and Silove, 2011).

The “back translation” method is another common tool used for developing proper measures across populations (Heine, 2012). Meffert et al. (2010) used this method, as well as Westermeyer and Uecker (1997) in a study on hostile responses among Hmong refugees. While useful, back translation does not improve conceptual frameworks in measurement tools, as nuances in ethno-behavioral descriptions of anger and trauma (e.g., individualistic and collectivist perspectives) are sometimes overlooked (cf. Hinton et al., 2003, 2009; Horton, 2006). The need for sensitivity to these models has been highlighted in studies on Bosnian and Kosovoan refugees (Ekblad et al., 2002; Summerfield, 2003; cf. Van de Vijver and Tanzer, 2004). In Summerfield's client's local language, for example, the only phrase that equates to “trauma” is “spiritual bruise,” which is a “…much wider and more holistic concept of injury than a scientifically framed one” (Summerfield, 2003). Furthermore, anger behavior among Kosovoan refugees in Sweden (*n* = 131) was correlated with difficulty in managing and comprehending verbal, emotional, and existential responses to experiences of torture (Ekblad et al., 2002; c.f. Antonovsky, 2013, cited in p. 32). However, Ekblad, Prochazka and Roth conceded that self-report measurements were too individualized and were, therefore, inappropriate for this particular sample.

Other researchers have aimed to explore the cross-cultural applicability of diagnostic models, such as the IED, a DSM disorder revolving around impulse control (Rees and Silove, 2011; Liddell et al., 2013). On the one hand, a clinical concordance study on a sample of Timorese IDPs (*n* = 85) showed high convergence (AUC index = 0.90) between both community-based and DSM-IV definitions of IED (Liddell et al., 2013). On the other hand, a qualitative study on West Papuan refugees (*n* = 41) uncovered a local idiom, “Sakit Hati,” which involves a chronic tendency to fixate on experiences of persecution, specifically, by the Indonesian army, frequently resulting in “explosive” anger responses (Rees and Silove, 2011). Though very similar to IED, the authors concluded that “Sakit Hati” emerges as a primarily affective anger behavior.

#### Ethno-behavior Science

Researchers should also be aware of ethno-behavioral perspectives on anger because they may influence how individuals report on, evaluate, or cope with it. For example, in Horton's (2006) qualitative study, a sample of Tibetan refugees (*n* = 109) disapproved of anger behavior, reporting on shorter and less intense anger episodes than a sample of Americans (*n* = 41). Likewise, Raney (2008), in a qualitative study on Tibetan refugee women (*n* = 12) in New York City, asked how participants felt about being forced to flee Tibet and having to resettle. Women narrated experiences of loss, terror, and isolation, but did not report feelings of anger.

Similar findings emerged from qualitative interviews with Buddhist monks (*n* = 6) from whom members of a USA-based Cambodian refugee community sought advice (Nickerson and Hinton, 2011). Reportedly, one third of this sample discouraged ruminating or feeling angry about injustices that had taken place under the Khmer Rouge Regime because doing so, according to Khmer belief, causes an upsurge of steam in the body resulting in cardiac arrest, also known as a “khyal attack” (Herbst, 1992; Hinton et al., 2003, 2009). Correspondingly, Hinton et al. (2009) found that 70% of a clinical sample (*n* = 68) of Khmer refugees resettled in the USA believed their anger would lead to such an attack.

#### Ethno-physiology

Lack of social acceptance for anger behavior in both Tibetan and Khmer cultures may have led to response bias in the above studies. However, an “ethno-*physio*logical” account of the above Khmer refugee community led Hinton et al. (2003, 2009) to hypothesize that that the Khmer fear of anger sets off a process of interoceptive conditioning. For this particular group, Hinton et al. (2003) proposed a cyclical mechanism for anger-triggered panic attacks, in which culturally-based verbal fears of biobehavioral anger responses will reinforce fear responses further. This hypothesis was supported by an earlier study on another clinical sample (*n* = 100), more than half of which condemned and avoided biobehavioral anger responses and, as a result, suffered panic attacks (Hinton et al., 2003). To further test this relationship, analysis of data from a somewhat larger sample (*n* = 143) revealed that anger-associated catastrophic cognitions explained high PTSD variance (54%). Clearly, cultural rules influence how individuals interpret biobehavioral anger responses, which then determines the course of subsequent responses. Again, such findings point to the importance of cultural sensitivity in research (cf. Betancourt et al., 2003, and Hinton et al., 2012).

### Social Consequences

While theories linking dysfunctional anger behavior among forced migrants and conflict on the meso and macro-level exist (e.g., see “etiology of terrorism;” Victoroff, 2005; Rice, 2009; Victoroff et al., 2012), our search uncovered correlations in the private sphere (c.f. Silove, 1999; Momartin and Coello, 2006; Hinton et al., 2009; Isakson and Layne, 2009; Silove et al., 2009; Brooks et al., 2011; Nickerson and Hinton, 2011; Bryant and Nickerson, 2013; Meffert et al., 2014). For example, maladaptive anger responses potentially contribute to self-injurious behavior among torture survivors (Momartin and Coello, 2006; Bryant and Nickerson, 2013), as well as family conflict, especially when acculturation gaps occur between generations (Hinton et al., 2009; Isakson and Layne, 2009; Nickerson and Hinton, 2011).

According to Silove et al. (2009), these negative social consequences may worsen especially when refugees have little means to improve their circumstances or advocate for social justice. Correspondingly, Meffert et al. (2014) theorize that PTSD-linked anger responses increase interpersonal conflict amongst populations living in broken, disempowered communities. Interestingly, problematic anger responses have decreased, in cases where individuals felt empowered through social support or were committed to and involved in their social causes (e.g., campaigns for restitution, migrant rights; Kira et al., 2009; Rees and Silove, 2011; cf. Zarowsky, 2004).

### Health Factors

Despite prosocial benefits, the biobehavioral aspect of anger responses is a risk factor for health, especially cardiovascular health (Tuomisto et al., 2005; Smith et al., 2013). In the refugee context, a study on Iraqi refugees (*n* = 501) resettled in the USA revealed that anger (i.e., refusal to forgive Saddam Hussein's collaborators) predicted hypertension, as well as digestive and respiratory problems (Kira et al., 2009). In the same sample, however, anger (i.e., refusal to forgive Saddam Hussein himself) predicted a significant decrease in circulatory disorders. Kira et al. (2009) posited that shared, righteous anger gave the participants a sense of control, belonging, and validation, suggesting that health effects of anger behavior seem to be dependent on social contingencies (i.e., if the behavior is negatively vs. positively reinforced). These results point to the importance of psychosocial support for treatment and intervention.

### Current Treatments and Interventions

#### Behavioral Interventions

It appears that Cognitive-Behavior Therapy (CBT) is the most evidence-based intervention for problematic anger responses that arise from experiences of forced migration (Hinton et al., 2012; Nickerson et al., 2015b). However, hardly any evidence exists as to which form of CBT is the most appropriate for individuals with refugee backgrounds (R. Bryant, personal communication, October 3, 2013). On the one hand, exposure treatment, especially live exposure, is proven most effective for clients who suffer from PTSD (Paunovic and Öst, 2001; Mineka and Zinbarg, 2006). An additional benefit is that exposure therapy is simplest for paraprofessionals to learn and is most easily disseminated across mass groups of people. On the other hand, clients often resist the regimen required for success (Sharp et al., 2004; Hinton et al., 2012), which may account for the 50% failure rate (Salcioglu and Başoglu, 2013).

Although past research on war veterans suggests that high levels of anger behavior inhibit successful exposure-based treatment outcomes for PTSD (Forbes et al., 2008), Stenmark et al. (2014) found that it did not affect outcomes among a clinical sample of refugees (*n* = 54). Nevertheless, Hinton et al. (2012) recommend that stretching and mindfulness exercises follow sessions, so that the client learns to associate positive sensations with anger responses, instead of unpleasant ones. According to Momartin and Coello (2006), torture survivors especially benefit from supplementary exercises like these.

#### Complicated Grief

Although exposure therapy is proven successful in treating prolonged bereavement, its efficacy in treating complicated bereavement may actually rely upon verbal processes, unlike treatment for PTSD, which teaches clients to regulate and tolerate anger responses (R. Bryant, personal communication, October 3, 2013). Therefore, therapists should explore alternative instructional interventions, as clients tend to resist exposure. Although further research is needed, instructional interventions may teach clients to re-evaluate anger responses and learn new ones, thus preventing rumination.

#### Cultural Sensitivity

No matter which form or combination of CBT is used, it is important for behavior therapists to increase cultural sensitivity and awareness of how cultural backgrounds influence anger responses (Hinton et al., 2012). For example, Nickerson and Hinton (2011) recommend that Acceptance and Commitment Therapy (ACT) be used in combination with techniques tailored to the individual's unique needs and cultural values.

#### Systems-Based and Multidisciplinary Approaches

Salcioglu and Başoglu (2013) advise against the use of multidisciplinary programs for treating PTSD. Although, Meffert et al. (2014) reported that Interpersonal Psychotherapy reduced post-traumatic anger responses in a randomized pilot study on Sudanese refugees (*n* = 22) in Cairo, the small sample size made it difficult to assess the significance of the study, thereby signifying the need for further research.

Nevertheless, the reviewed studies establish that problematic anger responses are also compounded by post-migration factors (see for example, Brooks et al., 2011; Nickerson et al., 2015b). Therefore, it may be necessary to address interpersonal dynamics of anger behavior, which are impacted by social and economic variables in post-migration contexts (cf. Rees et al., 2013). For instance, Hinton et al. (2009) recommend that education on post-traumatic stress responses and promotion of “cultural esteem” involve the whole family. Furthermore, policies and community-based programs promoting integration and civic participation are necessary to alleviate maladaptive anger responses and promote prosocial behavior (Rees et al., 2013).

## Discussion

Overall, experiences involving persecution, nationwide trauma, and multiple losses may lead to complex anger responses, further compounded by hostile post-migration environments (e.g., see Westermeyer and Uecker, 1997; Silove et al., 2009; Meffert et al., 2010; Brooks et al., 2011; Nickerson et al., 2015b). Therefore, diagnostic intervention should transcend conventionally framed PTSD models (cf. Silove, 1999, 2013) and, furthermore, focus on affect, rather than impulse control (cf. Rees and Silove, 2011).

Salcioglu and Başoglu (2013) recommend control-focused behavior therapy (C-FBT), instead of traditional exposure treatment, because it teaches clients to accept, rather than reduce, anger responses, and to acquire a “sense of control” over these responses. Accordingly, both C-FBT and traditional exposure treatment should involve training that increases the client's ability to adjust anger behavior in response to unpredictable circumstances (Bryant, personal communication, October 3, 2013; cf. Hinton et al., 2012).

Although Salcioglu and Başoglu (2013) claim that C-FBT has been validated across many samples of asylum seekers and refugees, a meta-analysis by Fuchs et al. (2013) maintains that ACT is the preferred method of intervention among clients originating from minority and marginalized backgrounds because of its humanistic, value-aware, and collaborative approach. Therefore, ACT not only draws upon the benefits of exposure therapy, but also provides a platform for clients to explore culturally-contingent thoughts and ideas without judgment (Eifert et al., 2006; Fuchs et al., 2013). Further research is needed to assess the validity of ACT across more refugee populations (Fuchs et al., 2013; see also Gardner and Moore (2013) on Contextual Anger Regulation Therapy).

Behavior analytic research involving multiple baseline designs (MBDs) will be especially useful for assessing the efficacy of interventions such as C-FBT and ACT (cf. Hawkins et al., 2007 and Hinton et al., 2003). As demonstrated in Hinton's (2003) study, MBDs are not only statistically reliable; they are also more practical and cost-effective than randomized controlled trials because they can be drawn from fewer sample sizes, making them ideal for epidemiological research (Hawkins et al., 2007).

### ADAPT Model

Certainly, maladaptive and adaptive anger responses are not “mutually exclusive” (cf. Silove, 2013), because they are contextually determined. Problematically, DSM and ICD constructs may be too rigid to account for the range of anger responses that follow experiences of forced migration, which lie on a spectrum of normal and problematic behavior (cf. Silove, 1999, 2013). Alternatively, the ADAPT model allows researchers and practitioners to explore anger responses as contingent upon complex, constantly changing environmental variables.

## Implications and Future Considerations

### Strengths and Limitations

This review's major strength lie in its novelty, scope, in-depth analysis, and detailed recommendations for future research. The review is also very timely as forced migration is ever increasing (e.g., because of population growth, climate change and following conflicts). This review, however, is limited in its reliance on relatively few studies to choose from overall. More research is needed to explore this topic, but a large quantitative review is not yet adequate because of limited research in the area.

Further epidemiological, cross-sectional, and longitudinal research, as well as “bottom-up” and single-case research designs, will be necessary to continue testing the ADAPT model and to begin the process of assessing its cross-cultural coherence in other refugee populations (e.g., see Hinton et al., 2003). Furthermore, Rees et al. (2013) recommend mixed methodology for exploring the social precipitants, contingencies, consequences of, and causal pathways between prosocial and problematic anger responses (cf. Brooks et al., 2011; cf. Kira et al., 2009; cf. Rees and Silove, 2011; cf. Zarowsky, 2004). As anger behavior is also a societal issue, avenues for reconciliation, expression of grievances, employment, civic participation, and integration are needed.

## Author Contributions

MT planned the study, analyzed the data, and wrote a part of the manuscript. JR planned the study, collected the data, analyzed the data, and wrote a part of the manuscript.

### Conflict of Interest Statement

The authors declare that the research was conducted in the absence of any commercial or financial relationships that could be construed as a potential conflict of interest.

## Footnotes

^1^As defined by the United Nations (UN), a refugee is “[a] person who owing to a well-founded fear of being persecuted for reasons of race, religion, nationality, membership of a particular social group or political opinion, is outside the country of his nationality and is unable or…unwilling to return to it” (UN General Assembly, 1951). For the purposes of this article, however, the authors herein use the term ‘refugee’ as an inclusive descriptor for all individuals with forced migration backgrounds (e.g., asylum seekers, Internally Displaced Persons (IDPS), those with Temporary Humanitarian Protection, Stateless Persons), unless stated otherwise.

^2^As defined by UNHCR (2011), the UN Refugee Agency, “an asylum seeker is an individual who is seeking international protection [and] whose claim has not yet been finally decided on by the country in which he or she has submitted it.”

^3^As defined by UNHCR, IDPs are persons within the borders of their own countries who have been forced to flee their homes to escape conflict, war, human rights violations, or humanitarian disasters.
